# Handgrip strength and risk of cognitive impairment across different glucose metabolism statuses: insights from the CHARLS study

**DOI:** 10.3389/fnagi.2025.1566652

**Published:** 2025-04-28

**Authors:** Shiqi Wang, Liangchen Li, Jia Yu, Xianli Sun, Jianqiao Chen

**Affiliations:** ^1^Department of General, Zhengzhou First People’s Hospital, Zhengzhou, China; ^2^Department of Traditional Chinese Medicine, Navy Qingdao Special Service Recuperation Center, Qingdao, China; ^3^Haikou Cadre’s Sanitarium of Hainan Military Region, Haikou, Hainan, China; ^4^Department of Health Medicine, Hainan Branch of General Hospital of Chinese PLA, Sanya, Hainan, China; ^5^Department of Geriatric, Henan Provincial People’s Hospital, People’s Hospital of Zhengzhou University, People’s Hospital of Henan University, Zhengzhou, China

**Keywords:** handgrip strength, cognitive impairment, glucose metabolism status, middle-aged and older, CHARLS

## Abstract

**Background:**

Both low handgrip strength (HGS) and abnormal glucose metabolism have been implicated in an increased risk of cognitive impairment. However, whether HGS interacts with glucose metabolism status to influence cognitive function remains unclear. This study explores the relationship between HGS and cognitive impairment risk among middle-aged and older Chinese adults and examines the potential modulation of this association by glucose metabolism status.

**Methods:**

Data from the China Health and Retirement Longitudinal Study (CHARLS) collected in 2011 and 2018 were analyzed, including 7,301 participants aged ≥ 45 years. Cognitive impairment was the primary outcome. Logistic regression and restricted cubic spline (RCS) analyses were applied to evaluate the association between HGS and cognitive impairment risk across different glucose metabolism statuses.

**Results:**

The study included 7,301 participants (mean age: 58.8 ± 8.9 years; 49.3% female). Over a 7-year follow-up, the mean cognitive function score declined from 12.05 ± 3.30 to 7.75 ± 5.70. After adjusting for confounders, logistic regression analyses indicated that higher HGS was significantly associated with a lower risk of cognitive impairment. Participants in the highest HGS quartile (Q4) had a significantly reduced odds of cognitive impairment compared to those in the lowest quartile (Q1) (odds ratio [OR]: 0.59, 95% confidence interval [CI]: 0.49–0.71; *P* < 0.001). RCS analysis demonstrated a significant negative linear correlation between HGS and cognitive impairment across individuals with normal glucose regulation, prediabetes, and diabetes (*P* < 0.001). The interaction *p*-value was 0.277, indicating no significant differences in this association among glucose metabolism subgroups.

**Conclusion:**

Higher HGS is significantly associated with a reduced risk of cognitive impairment among middle-aged and older individuals, irrespective of glucose metabolism status. These findings suggest that HGS assessment could be a valuable universal tool for evaluating cognitive impairment risk, regardless of metabolic conditions.

## 1 Introduction

With the intensification of global aging trends, cognitive diseases in middle-aged and older adults have emerged as critical medical and societal challenges. Cognitive impairment arises from damage to brain regions associated with cognitive functions, resulting in declines in memory, reasoning, and comprehension. Key symptoms include memory loss, reduced learning capacity, diminished attention span, and impaired motor coordination (Zhou et al., 2020). According to the World Health Organization, cognitive impairment represents a substantial threat to healthy aging among middle-aged and older populations (Ping et al., 2020). Given the insidious onset and low diagnostic rates of cognitive impairment, many individuals are diagnosed only at the dementia stage. Thus, identifying accessible and measurable risk indicators for early detection is paramount to enable timely intervention strategies.

Handgrip strength (HGS) is a well-established marker of muscle strength and overall physical fitness, particularly reflecting upper limb muscle function (Alencar et al., 2012; Bodilsen et al., 2015; Goins et al., 2011; Guerra and Amaral, 2009; Sanderson and Scherbov, 2014). As muscle strength is closely linked to general physical health, its decline can indicate broader health issues, including cognitive impairment. Although most studies have found a link between low HGS and an increased risk of cognitive decline and dementia, a small number of studies, including those in older adults, have reported no association between baseline HGS and cognitive decline (Camargo et al., 2016; Poon et al., 2022; Taekema et al., 2012; Vancampfort et al., 2019). These conflicting findings highlight the uncertainty surrounding the use of HGS as a reliable risk indicator for cognitive impairment. Furthermore, much of the existing evidence originates from studies conducted in developed Western countries, with limited population-based data on the association between HGS and cognitive function in middle-aged and older adults in Chinese communities.

The relationship between HGS and cognitive function may be influenced by underlying health conditions, particularly glucose metabolism status. Epidemiological evidence shows that individuals with diabetes mellitus (DM) are at significantly higher risk of cognitive impairment compared to those with normal glucose regulation, with 60–70% of DM patients experiencing varying degrees of cognitive deficits. These deficits include memory loss, decreased attention and executive function, and impaired motor coordination, which may progress to dementia (Biessels et al., 2006; Cukierman-Yaffe et al., 2009; McCrimmon et al., 2012). Given the strong association between glucose metabolism disorders and cognitive decline, it is important to examine how HGS is related to cognitive impairment in individuals with different glucose metabolism statuses. Exploring this relationship across different glucose metabolism statuses is critical for developing targeted management strategies for distinct populations.

Therefore, using data from the China Health and Retirement Longitudinal Study (CHARLS), this study examines the association between HGS and cognitive impairment among middle-aged and older adults across different glucose metabolism statuses. By providing robust population-based evidence, it aims to contribute to the scientific understanding of HGS as a risk indicator and to support clinical early intervention and risk prediction strategies for cognitive impairment in populations with varying glucose metabolism profiles.

## 2 Materials and methods

### 2.1 Study population

**FIGURE 1:**
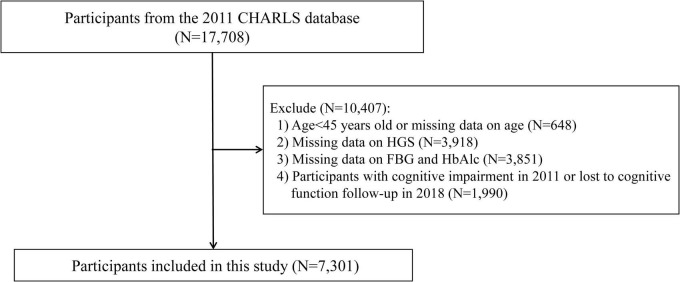
Study flowchart. CHARLS, China Health and Retirement Longitudinal Study; FBG, fasting blood glucose; HbAlc, glycated hemoglobin; HGS, handgrip strength.

The CHARLS is a nationwide longitudinal cohort targeting middle-aged and older adults (≥ 45 years) in China, designed to collect extensive data on demographics, health status, economic factors, and social conditions (Zhao et al., 2014). For the present analysis, we utilized data from the 2011 (baseline) and 2018 waves of the CHARLS dataset. The initial cohort comprised 17,708 participants. To ensure data integrity and suitability for analysis, we applied the following exclusion criteria: (1) participants younger than 45 years or those with missing age data (*n* = 648); (2) participants with missing data on HGS (*n* = 3,918) or fasting blood glucose (FBG) and glycated hemoglobin (HbA1c) (*n* = 3,851); and (3) participants with cognitive impairment at baseline (2011) or those lost to follow-up for cognitive function assessment in 2018 (*n* = 1,990). Following these exclusions, 7,301 participants were retained for the final analysis (Figure 1).

The CHARLS protocol was approved by the Institutional Review Board of Peking University (IRB00001052-11015). Written informed consent was obtained from all participants before enrollment.

### 2.2 Measurement of cognitive function and HGS

Cognitive function was evaluated using a comprehensive battery of assessments, including episodic memory, the 10-item Telephone Interview for Cognitive Status (TICS) (Fong et al., 2009), and a figure-drawing task. Episodic memory was assessed by asking participants to recall a list of 10 Chinese nouns immediately after hearing them (immediate recall) and again after a four-minute delay (delayed recall). The average of the immediate and delayed recall scores (range: 0–10) was computed. The TICS included 10 questions evaluating temporal orientation (e.g., identifying the current season and date) and cognitive performance, such as serial subtraction of 7 from 100, repeated up to five times. Additionally, participants were shown an image of two overlapping pentagons and were instructed to replicate it (scored as 0 = failure or 1 = success). The total cognitive function score, ranging from 0 to 21, was calculated by summing the results of these three tests. This methodology has been validated in prior CHARLS studies (Li et al., 2017). Cognitive impairment was defined as a total score at least 1.0 standard deviation (SD) below the mean cognitive function score, consistent with established literature (Bai et al., 2021; Jak et al., 2009).

HGS was measured using a mechanical dynamometer (Yuejian WL-1000, Nantong, China) by trained personnel (Zhao et al., 2014). Referring to the Asian Working Group for Sarcopenia 2019 consensus guidelines, participants performed two maximum-force trials for each hand with maximal effort, and the highest HGS value from the dominant hand was used for analysis (Chen et al., 2020). Based on quartile distribution, participants were stratified into four groups (Q1, Q2, Q3, and Q4) according to their HGS index.

### 2.3 Covariates

To mitigate potential confounding effects, the analysis incorporated the following covariates: demographic factors (age and gender); sociodemographic factors (body mass index [BMI], marital status, residence, education level, smoking status, and drinking status); medical history (health status, presence of chronic diseases, and hypertension); laboratory parameters (low-density lipoprotein cholesterol [LDL-C], total cholesterol [TC], FBG, and HbA1c); cognitive function in 2011.

### 2.4 Statistical analysis

Descriptive statistics were employed to summarize the dataset. Data were reported as mean ± standard deviation (SD), median (interquartile range), or frequency and percentage, depending on the variable type. Categorical variables were compared using chi-square tests, while continuous variables were analyzed using one-way analysis of variance (ANOVA) or the Kruskal–Wallis test for data not normally distributed.

To assess the association between HGS and cognitive impairment, logistic regression analyses were conducted, with results expressed as adjusted odds ratios (OR) and 95% confidence intervals (CI). Three logistic regression models were constructed: Model 1 included univariate logistic regression; Model 2 adjusted for age and gender; and Model 3 incorporated additional adjustments for age, gender, BMI, marital status, residence, education level, health status, smoking status, drinking status, chronic diseases, hypertension, LDL-C, TC, FBG, HbA1c, and cognitive function in 2011. Restricted cubic spline (RCS) regression was utilized to examine the relationship between HGS levels and cognitive impairment across different glucose metabolic statuses, namely normal glucose regulation (NGR), prediabetes (Pre-DM), and DM. HGS quartiles served as the reference category. The RCS analysis was further applied to elucidate the association between baseline HGS levels and the risk of cognitive impairment within these metabolic subgroups.

Subgroup analyses were conducted to investigate the association between HGS and cognitive impairment risk across strata defined by age (45–60 years and ≥ 60 years), gender (male and female), BMI (< 24 kg/m^2^ and ≥ 24 kg/m^2^), residence (urban and rural), and hypertension status (presence or absence). All statistical analyses were performed using R software version 4.2.2 (R Foundation for Statistical Computing, Vienna, Austria). Statistical significance was set at a two-sided *p*-value < 0.05.

## 3 Results

### 3.1 Baseline characteristics of study participants

Table 1 presents the baseline characteristics of the study participants, stratified by HGS quartiles. The study included 7,301 participants with a mean age of 58.8 ± 8.9 years, of whom 49.3% were female (*n* = 3,601). Participants were classified into four groups according to HGS quartiles: Q1 (≤ 26 kg), Q2 (> 26 kg and ≤ 33 kg), Q3 (> 33 kg and ≤ 40 kg), and Q4 (> 40 kg). The mean baseline HGS was 33.70 ± 10.16 kg. The majority of participants resided in rural areas (61.9%) and were married (89.3%), while 40.9% had no formal education. Over the 3-year follow-up period, the mean cognitive function score was 7.75 ± 5.70. Significant differences in baseline characteristics were observed across the HGS quartiles, including age, gender, BMI, marital status, educational attainment, health status, smoking and drinking habits, presence of hypertension, dyslipidemia, coronary heart disease (CHD), chronic diseases, lipid profiles (total cholesterol [TC], triglycerides [TG], high-density lipoprotein cholesterol [HDL-C], and LDL-C), HbA1c, and cognitive function scores (all *P* < 0.05). Detailed baseline characteristics are provided in Table 1.

**TABLE 1:** Baseline characteristics of participants categorized by HGS quartiles.

Characteristic	Overall (*n* = 7,301)	Q1 (≤ 26 kg) *n* = 1,822	Q2 (26, 33 kg) *n* = 1,805	Q3 (33, 40 kg) *n* = 1,948	Q4 (> 40 kg) *n* = 1,726	*P*-value
Age, mean ± SD, years	58.8 ± 8.9	61.8 ± 9.2	59.0 ± 9.2	58.5 ± 8.6	55.5 ± 7.2	< 0.001
Female, *n* (%)	3,601 (49.3)	1,570 (86.2)	1,264 (70.0)	649 (33.3)	118 (6.8)	< 0.001
**BMI, kg/m^2^**						< 0.001
< 24	4,153 (57.4)	1,065 (59.3)	1,006 (56.1)	1,159 (60.0)	923 (53.8)	
≥ 24	3,085 (42.6)	732 (40.7)	787 (43.9)	772 (40.0)	794 (46.2)	
**Marital status, *n* (%)**						< 0.001
Married	6,519 (89.3)	1,497 (82.2)	1,600 (88.6)	1,782 (91.5)	1,640 (95.0)	
Others	782 (10.7)	325 (17.8)	205 (11.4)	166 (8.5)	86 (5.0)	
**Residence, *n* (%)**						0.372
Rural	4,520 (61.9)	1,108 (60.8)	1,133 (62.8)	1,227 (63.0)	1,052 (61.0)	
Urban	2,781 (38.1)	714 (39.2)	672 (37.2)	721 (37.0)	674 (39.0)	
**Education level, *n* (%)**						< 0.001
No formal education	2,986 (40.9)	1,052 (57.7)	859 (47.6)	680 (34.9)	395 (22.9)	
Primary school	1,779 (24.4)	398 (21.8)	447 (24.8)	525 (27.0)	409 (23.7)	
Middle or high school	1,689 (23.1)	260 (14.3)	342 (19.0)	495 (25.4)	592 (34.3)	
College or above	842 (11.5)	112 (6.1)	155 (8.6)	247 (12.7)	328 (19.0)	
**Health, *n* (%)**						< 0.001
Poor	260 (3.6)	99 (5.4)	64 (3.5)	71 (3.6)	26 (1.5)	
Fair	1,558 (21.3)	531 (29.2)	405 (22.5)	367 (18.8)	255 (14.8)	
Good	3,809 (52.2)	888 (48.8)	977 (54.1)	1,047 (53.9)	897 (52.0)	
Very good and above	1,672 (22.9)	303 (16.6)	359 (19.9)	462 (23.7)	548 (31.7)	
**Smoking status, *n* (%)**						< 0.001
Never or former	4,927 (67.5)	1,573 (86.3)	1,424 (78.9)	1,158 (59.4)	772 (44.7)	
Current	2,373 (32.5)	249 (13.7)	380 (21.1)	790 (40.6)	954 (55.3)	
**Drinking status, *n* (%)**						< 0.001
Never or former	4,747 (65.0)	1,532 (84.1)	1,392 (77.1)	1,134 (58.2)	689 (39.9)	
Current	2,554 (35.0)	290 (15.9)	413 (22.9)	814 (41.8)	1,037 (60.1)	
Hypertension, *n* (%)	3,505 (48.1)	947 (52.2)	870 (48.2)	896 (46.1)	792 (45.9)	< 0.001
Dyslipidemia, *n* (%)	3,783 (51.8)	981 (53.9)	956 (53.0)	969 (49.8)	877 (50.8)	0.046
CHD, *n* (%)	912 (12.5)	286 (15.7)	236 (13.1)	237 (12.2)	153 (8.9)	< 0.001
Stroke, *n* (%)	176 (2.4)	52 (2.9)	45 (2.5)	45 (2.3)	34 (2.0)	0.383
Chronic diseases, *n* (%)	5,088 (69.7)	1,389 (76.2)	1,264 (70.0)	1,361 (69.9)	1,074 (62.2)	< 0.001
TC, mean ± SD, mg/dl	193.53 ± 38.13	196.82 ± 38.10	195.16 ± 38.74	191.31 ± 36.78	190.87 ± 38.68	< 0.001
TG, median (IQR), mg/dl	107.05 (75.23, 156.66)	113.12 (79.71, 160.69)	106.73 (76.15, 153.21)	102.33 (72.62, 150.82)	106.75 (74.32, 161.74)	< 0.001
HDL-C, mean ± SD, mg/dl	50.71 ± 15.13	51.30 ± 14.52	51.68 ± 15.27	50.68 ± 15.16	49.12 ± 15.45	< 0.001
LDL-C, mean ± SD, mg/dl	116.48 ± 34.78	119.20 ± 34.98	117.48 ± 34.81	114.87 ± 34.03	114.37 ± 35.15	< 0.001
FBG, mean ± SD, mg/dl	110.38 ± 37.25	112.28 ± 41.86	110.18 ± 38.21	109.44 ± 34.42	109.64 ± 33.96	0.081
HbAlc, mean ± SD,%	5.26 ± 0.80	5.32 ± 0.93	5.27 ± 0.82	5.22 ± 0.72	5.23 ± 0.72	0.001
Cognitive function in 2011, mean ± SD	12.05 ± 3.30	10.86 ± 3.47	11.72 ± 3.31	12.38 ± 3.10	13.29 ± 2.80	< 0.001
Cognitive function in 2018, mean ± SD	7.75 ± 5.70	6.30 ± 5.46	7.37 ± 5.58	7.90 ± 5.74	9.49 ± 5.58	< 0.001
**GMS, *n* (%)**						0.152
NGR	3,032 (41.5)	757 (41.5)	753 (41.7)	801 (41.1)	721 (41.8)	
Pre-DM	3,171 (43.4)	755 (41.4)	786 (43.5)	874 (44.9)	756 (43.8)	
DM	1,098 (15.0)	310 (17.0)	266 (14.7)	273 (14.0)	249 (14.4)	

BMI, body mass index; CHD, coronary heart disease; DM, diabetes mellitus; FBG, fasting blood glucose; GMS, glucose metabolic states; HbAlc, glycated hemoglobin; HDL-C, high-density lipoprotein cholesterol; HGS, handgrip strength; IQR, inter quartile range; LDL-C, low-density lipoprotein cholesterol; NGR, normal glucose regulation; Pre-DM, prediabetes mellitus; Q, quartile; SD, standard deviation; TC, total cholesterol; TG, triglycerides.

### 3.2 Association between HGS and cognitive impairment

**FIGURE 2:**
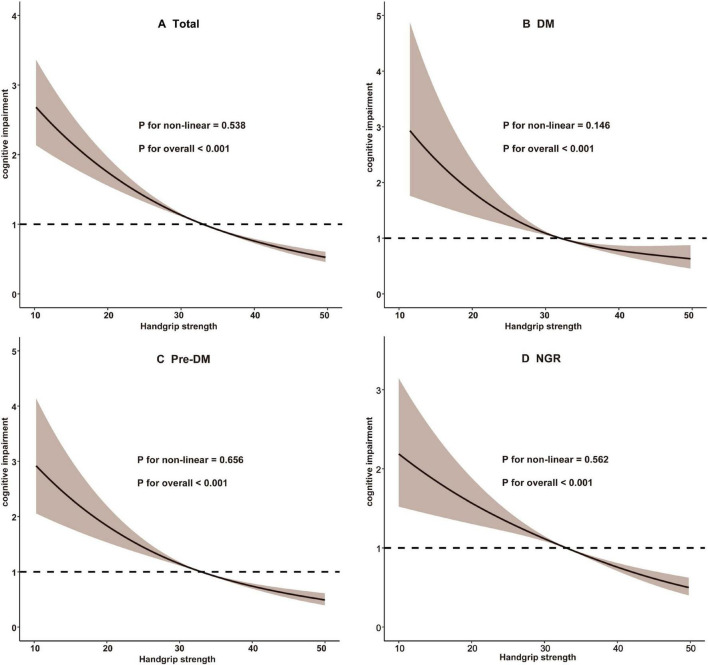
Restricted cubic spline analysis of the association between handgrip strength and cognitive impairment. **(A)** Total participants; **(B)** Participants with DM; **(C)** Participants with Pre-DM. **(D)** Participants with NGR. DM, diabetes mellitus; NGR, normal glucose regulation; Pre-DM, prediabetes.

Cognitive function assessments conducted in 2011 yielded a mean score of 12.05 ± 3.30, which declined to 7.75 ± 5.70 during the follow-up period in 2018. After adjusting for potential confounders, a significant association was identified between HGS quartiles and the risk of cognitive impairment. Higher HGS quartiles were associated with a reduced risk of cognitive impairment, with statistical significance maintained across all three analytical models (*P* for trend < 0.001). In Model 3, participants in the highest HGS quartile (Q4) exhibited a significantly lower risk of cognitive impairment compared to those in the lowest quartile (Q1), with an OR of 0.59 (95% CI: 0.49–0.71, *P* < 0.001) (Table 2). RCS analysis further demonstrated a linear relationship between HGS and cognitive impairment risk (overall *P* < 0.001; non-linear *P* = 0.538) (Figure 2).

**TABLE 2:** The association between HGS and the risk of cognitive impairment.

Categories	Event, *n* (%)	Model 1^a^		Model 2^b^		Model 3^c^	
		**OR (95% CI)**	***P*-value**	**OR (95% CI)**	***P*-value**	**OR (95% CI)**	***P*-value**
Quartile 1	927 (50.9%)	Ref.		Ref.		Ref.	
Quartile 2	762 (42.2%)	0.71 (0.62–0.80)	< 0.001	0.85 (0.74–0.97)	0.019	0.87 (0.75–1.00)	0.051
Quartile 3	745 (38.2%)	0.60 (0.52–0.68)	< 0.001	0.76 (0.64–0.89)	< 0.001	0.79 (0.67–0.93)	0.004
Quartile 4	437 (25.3%)	0.36 (0.32–0.41)	< 0.001	0.57 (0.47–0.68)	< 0.001	0.59 (0.49–0.71)	< 0.001
*P* for trend			< 0.001		< 0.001		< 0.001

HGS, handgrip strength; CI, confidence interval; OR, odds ratio.

^a^Unadjusted model.

^b^Adjusted for age and gender.

^c^Adjusted for age, gender, body mass index, marital status; residence, education level, health status, smoking status, drinking status, chronic diseases, hypertension, low-density lipoprotein cholesterol, total cholesterol, fasting blood glucose, glycated hemoglobin, and cognitive function in 2011.

### 3.3 Association between HGS and cognitive impairment across glucose metabolic status

As summarized in Table 3, Model 3 revealed that participants in higher HGS quartiles were consistently associated with a reduced risk of cognitive impairment across all glucose metabolic status categories (NGR, Pre-DM, and DM). Specifically, in the NGR group, the ORs for cognitive impairment were 0.98 (95% CI: 0.79–1.23) for Q2, 0.82 (95% CI: 0.63–1.05) for Q3, and 0.60 (95% CI: 0.45–0.81) for Q4. Among Pre-DM participants, the ORs were 0.82 (95% CI: 0.66–1.02) for Q2, 0.78 (95% CI: 0.60–0.99) for Q3, and 0.59 (95% CI: 0.44–0.79) for Q4. For DM participants, the ORs were 0.76 (95% CI: 0.53–1.09) for Q2, 0.79 (95% CI: 0.52–1.19) for Q3, and 0.59 (95% CI: 0.37–0.95) for Q4. RCS analysis indicated a predominantly linear inverse association between HGS and cognitive impairment risk across all glucose metabolic status groups (NGR, *P*-non-linear = 0.562; Pre-DM, *P*-non-linear = 0.656; DM, *P*-non-linear = 0.146) (Figure 2). Furthermore, the interaction term (*P*-interaction = 0.277) suggested no significant differences in the relationship between HGS and cognitive impairment across glucose metabolism categories (Table 3).

**TABLE 3:** The association between HGS and the risk of cognitive impairment according to glucose metabolism status.

Categories	Event, *n* (%)	Model 1^a^		Model 2^b^		Model 3^c^		
		**OR (95% CI)**	***P*-value**	**OR (95% CI)**	***P*-value**	**OR (95% CI)**	***P*-value**	***P*-interaction**
**NGR**								0.277
Quartile 1	366 (48.3%)	Ref.		Ref.		Ref.		
Quartile 2	322 (42.8%)	0.80 (0.65–0.98)	0.029	0.98 (0.79–1.22)	0.862	0.98 (0.79–1.23)	0.894	
Quartile 3	296 (37.0%)	0.64 (0.52–0.79)	< 0.001	0.81 (0.63–1.04)	0.095	0.82 (0.63–1.05)	0.116	
Quartile 4	182 (25.2%)	0.39 (0.31–0.48)	< 0.001	0.60 (0.46–0.80)	< 0.001	0.60 (0.45–0.81)	< 0.001	
*P* for trend			< 0.001		< 0.001		< 0.001	
**Pre-DM**								
Quartile 1	386 (51.1%)	Ref.		Ref.		Ref.		
Quartile 2	324 (41.2%)	0.67 (0.55–0.82)	< 0.001	0.79 (0.64–0.97)	0.028	0.82 (0.66–1.02)	0.070	
Quartile 3	326 (37.3%)	0.56 (0.46–0.69)	< 0.001	0.72 (0.56–0.92)	0.009	0.78 (0.60–0.99)	0.047	
Quartile 4	182 (24.1%)	0.34 (0.28–0.42)	< 0.001	0.52 (0.39–0.69)	< 0.001	0.59 (0.44–0.79)	< 0.001	
*P* for trend			< 0.001		< 0.001		< 0.001	
**DM**								
Quartile 1	175 (56.5%)	Ref.		Ref.		Ref.		
Quartile 2	116 (43.6%)	0.60 (0.43–0.83)	0.022	0.71 (0.50–1.01)	0.060	0.76 (0.53–1.09)	0.136	
Quartile 3	123 (45.1%)	0.62 (0.43–0.87)	0.006	0.73 (0.49–1.10)	0.131	0.79 (0.52–1.19)	0.260	
Quartile 4	73 (29.3%)	0.37 (0.27–0.51)	< 0.001	0.62 (0.39–0.97)	0.038	0.59 (0.37–0.95)	0.031	
*P* for trend			< 0.001		0.003		0.001	

CI, confidence interval; DM, diabetes mellitus; HGS, handgrip strength; NGR, normal glucose regulation; OR, odds ratio; Pre-DM, prediabetes mellitus.

^a^Unadjusted model.

^b^Adjusted for age and gender.

^c^Adjusted for age, gender, body mass index, marital status; residence, education level, health status, smoking status, drinking status, chronic diseases, hypertension, low-density lipoprotein cholesterol, total cholesterol, fasting blood glucose, glycated hemoglobin, and cognitive function in 2011.

### 3.4 Subgroup analyses

**FIGURE 3:**
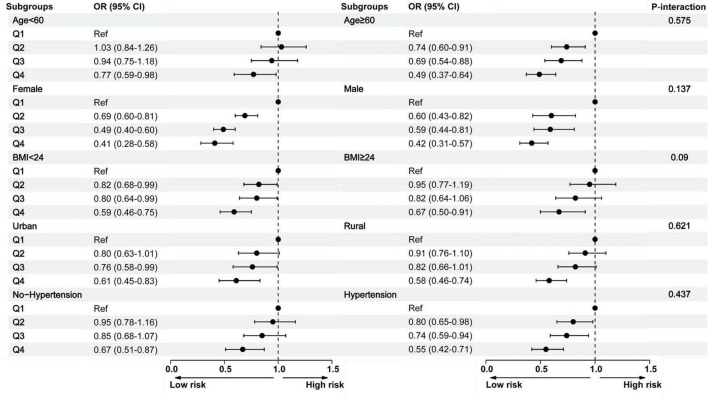
Subgroup analysis between the handgrip strength and cognitive impairment across various subgroups. BMI, body mass index; CI, confidence interval; Q, quartile; OR, odds ratio.

Subgroup analyses further demonstrated consistent findings across various stratified subgroups, including gender, age, BMI, residential setting (rural/urban), and hypertension status. No significant interaction effects were observed (*P*-interaction > 0.05). Across all subgroups, participants in the highest HGS quartile (Q4) consistently exhibited a significantly reduced risk of cognitive impairment (Figure 3 and Supplementary Table 1).

## 4 Discussion

This study identified a significant negative linear association between higher HGS and the risk of cognitive impairment in middle-aged and older adults. Importantly, this negative correlation was consistent across different glucose metabolism statuses, including NGR, Pre-DM, and DM. These findings underscore the potential of HGS as a simple and non-invasive biomarker for assessing the risk of cognitive impairment across populations with varying glucose metabolism statuses.

In alignment with prior research, cognitive impairment frequently coexists with diminished muscle strength in middle-aged and older adults, suggesting a strong interconnection between these factors (Cui et al., 2021; Kaczmarek et al., 2022). Several cross-sectional studies have consistently reported associations between reduced HGS and cognitive impairment (Bohannon, 2019; Li et al., 2018). However, while numerous epidemiological studies have explored the relationship between HGS and cognitive impairment, including those focusing on cognitive decline and dementia, the findings remain inconclusive (Camargo et al., 2016; Hatabe et al., 2020; Kim et al., 2019; Stessman et al., 2017; Veronese et al., 2016). Several studies conducted in older US populations have reported a significant association between lower HGS and an increased risk of cognitive impairment, suggesting that muscle strength may serve as an early marker of cognitive impairment (Hatabe et al., 2020; Kim et al., 2019; McGrath et al., 2019). In contrast, other studies have not observed such an association, implying that the relationship between HGS and cognitive impairment may be influenced by differences in study populations, methodologies, or underlying health conditions (Camargo et al., 2016; Stessman et al., 2017; Veronese et al., 2016). Furthermore, many of these studies utilized cross-sectional or case-control designs, which limit the ability to draw conclusions about temporal relationships (Ikegami et al., 2019; Narazaki et al., 2014).

Considering the reported association between HGS and cognitive impairment, it is important to consider whether this relationship is influenced by metabolic health, particularly DM. DM has been consistently linked to an elevated risk of cognitive impairment, with individuals with DM exhibiting a higher susceptibility to cognitive decline compared to those without DM (Biessels and Despa, 2018). The underlying mechanisms are multifaceted, involving chronic hyperglycemia, insulin resistance, cerebrovascular dysfunction, and neuroinflammation (Biessels and Despa, 2018; Zilliox et al., 2016). Among these, hyperglycemia has been identified as a key driver of neurodegenerative processes, as it exacerbates oxidative stress, promotes amyloid-beta accumulation, and accelerates neuronal apoptosis, all of which contribute to cognitive decline. Additionally, insulin resistance in the brain impairs synaptic plasticity and glucose metabolism, further compromising cognitive function (Biessels et al., 2006; Koekkoek et al., 2015). Despite these well-documented links between DM and cognitive impairment, limited research has explored whether metabolic dysfunction modifies the association between HGS and cognitive impairment. Given that both muscle strength and cognitive function are influenced by metabolic pathways, it is plausible that DM may alter the strength or direction of this association. However, whether HGS serves as an equally robust marker of cognitive impairment across different glucose metabolism statuses remains unclear. To address this gap, we stratified our analysis by glucose metabolism status to examine whether DM modifies the HGS-cognition relationship. Understanding this potential interaction is crucial for determining the generalizability of HGS as a clinical marker for cognitive impairment across metabolically diverse populations.

In this context, we analyzed data from 7,301 participants collected between 2011 and 2018, stratifying them by HGS levels. Our results demonstrate that higher HGS levels are progressively associated with a lower risk of cognitive impairment, with the inverse relationship being most pronounced in the third and fourth HGS quartiles. Stratified analyses reveal that in the NGR and Pre-DM groups, higher HGS quartiles consistently correspond to a reduced risk of cognitive impairment. Similarly, in the DM group, higher HGS quartiles are associated with a lower risk of cognitive impairment, although the magnitude of the association is relatively attenuated compared to the NGR and Pre-DM groups. Notably, the interaction *p*-value of 0.277 indicates no significant differences in the relationship between HGS and cognitive impairment risk across metabolic status groups. These findings suggest a robust inverse relationship between HGS and cognitive impairment risk that transcends glucose metabolism status, highlighting the potential utility of HGS as a risk marker in diverse populations. Consequently, regular HGS assessment and muscle-strengthening interventions may hold promise for mitigating cognitive impairment risk in middle-aged and older adults, irrespective of their glucose metabolism status.

The biological underpinnings of the observed relationship between HGS and cognitive impairment likely involve the nervous and motor systems, which play a central role in regulating HGS (Carson, 2018; McGrath et al., 2019). Age-related cognitive decline is closely linked to the deterioration of these systems (McGrath et al., 2019). One plausible mechanism is that HGS reflects the functional integrity of the nervous system (Alfaro-Acha et al., 2006). Cortical and subcortical brain regions that control HGS and grasping movements also regulate cognitive functions (Buchman and Bennett, 2011). Since cognitive impairment is associated with cumulative brain atrophy and white matter hyperintensity, greater muscle strength has been correlated with larger brain and white matter volumes and smaller white matter lesion volumes (Aribisala et al., 2013). HGS training has been shown to enhance local efficiency in white matter connectivity, potentially improving cognitive function. White matter remodeling may thus represent a physiological mechanism linking HGS to cognitive function (Shang et al., 2021). Moreover, both muscle strength decline and cognitive impairment share common pathophysiological mechanisms, including inflammation and oxidative stress. Greater muscle strength may confer resilience against oxidative stress and inflammatory responses, thereby supporting cognitive function.

Our findings demonstrated that the influence of HGS on the risk of cognitive impairment did not vary significantly across different glucose metabolism statuses. This observation may be explained by the presence of shared pathophysiological mechanisms underlying cognitive impairment, including microvascular dysfunction, neuroinflammation, and β-amyloid deposition, which likely exert similar effects regardless of glucose metabolism status. Moreover, the moderating role of glucose metabolism abnormalities in the relationship between HGS and cognitive function may be limited, or its effect may be overshadowed by overarching risk factors such as systemic inflammation, vascular health, and lifestyle factors. These results highlight the potential utility of HGS as a broadly applicable indicator for assessing cognitive impairment risk.

Our findings carry significant clinical relevance, highlighting the potential of HGS as a practical tool for cognitive risk assessment across different glucose metabolism statuses. As a simple, cost-effective, and non-invasive measure, HGS could be integrated into routine health evaluations to facilitate early identification of individuals at risk for cognitive impairment. With the increasing trend of cognitive impairment occurring at younger ages, particularly due to lifestyle-related risk factors, monitoring muscle strength in middle-aged and older adults may offer an opportunity for earlier intervention before significant cognitive decline occurs. Moreover, the consistent association between HGS and cognitive impairment across different glucose metabolism statuses underscores the importance of muscle strength assessment regardless of metabolic health. While DM accelerates cognitive decline, our findings suggest that reduced HGS remains a relevant indicator of cognitive risk even in individuals with normal glucose metabolism. Strengthening interventions, such as resistance training programs, may not only enhance physical function but also support cognitive health, offering potential benefits across diverse metabolic conditions. Future studies should explore whether specific DM-related complications, such as microvascular dysfunction or systemic inflammation, influence the effectiveness of these interventions in preserving cognitive function. Future studies should explore whether specific DM-related complications, such as microvascular dysfunction or systemic inflammation, influence the effectiveness of these interventions in preserving cognitive function. In addition, further studies are needed to determine the long-term impact of maintaining or improving muscle strength on cognitive trajectories across different glucose metabolism statuses. Another important area of investigation is whether a threshold effect exists, where cognitive decline accelerates once muscle strength falls below a critical level. Furthermore, integrating biomarkers of metabolic and vascular health into future research may help elucidate underlying mechanisms and identify individuals who would benefit most from muscle-strengthening interventions.

Several limitations of this study must be acknowledged. First, the sample was restricted to middle-aged and older adults in China aged 45 years and above, which may limit the generalizability of these findings to other populations. Future research should investigate the relationship between HGS and cognitive impairment risk in diverse demographic groups. Second, the study relied on baseline measurements of HGS and did not account for the potential impact of changes in HGS on cognitive function. Third, cognitive function was assessed using measures of episodic memory and mental acuity rather than clinical diagnoses, limiting the multidimensional perspective necessary for a comprehensive evaluation of cognitive function. Nevertheless, these neuropsychological tests are well-validated and strongly associated with cognitive impairment, rendering them appropriate for this study. Finally, although adjustments were made for some potential confounding variables, the influence of unmeasured factors cannot be entirely excluded. Future investigations should aim to address these limitations by exploring additional confounding variables and conducting more comprehensive studies in broader populations.

## 5 Conclusion

Using data from the CHARLS, this study systematically examined the association between HGS and the risk of cognitive impairment among middle-aged and older adults with varying glucose metabolism statuses. The findings reveal a significant inverse relationship between HGS and cognitive impairment risk, exhibiting a linear trend across the overall population and within glucose metabolism subgroups. Notably, glucose metabolism status did not significantly influence the strength of this association. These results highlight the utility of HGS as a straightforward, non-invasive measure with broad potential for assessing cognitive impairment risk across diverse populations. Future intervention studies should investigate the efficacy of enhancing muscle strength as a strategy to improve cognitive health, offering a promising avenue for the protection of cognitive function in middle-aged and older adults.

## Data Availability

Publicly available datasets were analyzed in this study. This data can be found here: The data is sourced from the publicly accessible CHARLS database (http://charls.pku.edu.cn/). The datasets used during the current study are available from the corresponding authors on reasonable request.
