# Supplementary material for: Handgrip strength and risk of cognitive impairment across different glucose metabolism statuses: insights from the CHARLS study
Source: Front Aging Neurosci. 2025 Apr 28;17:1566652. doi: 10.3389/fnagi.2025.1566652 (PMC12066481; doi:10.3389/fnagi.2025.1566652)
Supplement: Supplementary file 1 [file Table_1.DOCX]

**Supplementary Table 1** **Subgroup analysis of the association between handgrip strength and** **cognitive impairment** **by age group**

| Subgroups | handgrip strength | OR (95% CI) | *P* value | *P*-interaction |
| --- | --- | --- | --- | --- |
| **Age** | |  |  | 0.321 |
| **<60 years** | Q1 | Ref. |  |  |
|  | Q2 | 1.03 (0.84-1.26) | 0.786 |  |
|  | Q3 | 0.94 (0.75-1.18) | 0.573 |  |
|  | Q4 | 0.77 (0.59-0.98) | 0.044 |  |
|  |  |  |  |  |
| **≥60 years, <70 years** | Q1 | Ref. |  |  |
|  | Q2 | 0.81 (0.63-1.03) | 0.084 |  |
|  | Q3 | 0.85 (0.64-1.14) | 0.280 |  |
|  | Q4 | 0.55 (0.40-0.76) | <0.001 |  |
|  |  |  |  |  |
| **≥70 years** | Q1 | Ref. |  |  |
|  | Q2 | 0.55 (0.36-0.84) | 0.005 |  |
|  | Q3 | 0.42 (0.26-0.66) | <0.001 |  |
|  | Q4 | 0.37 (0.21-0.67) | <0.001 |  |

**Abbreviation:** CI: confidence interval; Q: quartile; OR: odds ratio.
